# The Effect of Early Deprivation on Mental Health and Cognition

**DOI:** 10.1002/alz70860_100989

**Published:** 2025-12-23

**Authors:** Morgane Kuenzi, Delia A Gheorghe, Abhaya Adlakha, Sarah Bauermeister

**Affiliations:** ^1^ University of Oxford, Oxford, United Kingdom; ^2^ Transylvanian Institute of Neuroscience, Cluj‐Napoca, Cluj, Romania; ^3^ University of Oxford, Oxford, Oxfordshire, United Kingdom

## Abstract

**Background:**

Dementia is one of the leading causes of death, and at least 55 million people worldwide currently have dementia (WHO). Understanding the factors that increase the risk of developing dementia is essential for prevention and development of interventions. Literature has demonstrated that experiencing deprivation in childhood has long‐lasting detrimental effects on cognition and mental health (a risk factor for developing dementia). Therefore, this study aims to investigate the effects of early deprivation on depression and cognition later in life and understand the mechanistic pathways that underlie these associations. Targeting interventions on risk factors supports healthy cognitive aging.

**Method:**

This study aims to investigate the effect of deprivation experienced in childhood on cognition and depression later in life. For this research, longitudinal data from the English Longitudinal Study of Ageing (ELSA) dataset are used, and Structural Equation Modeling (SEM) is applied (*N* = 9,749, Mdn_age_ = 70, SD_age_ = 8.69). Age, gender, and highest educational qualification achieved are used as covariates.

**Result:**

Data analyses are currently being undertaken. Preliminary results show that financial hardship and the number of books at home predicted higher depressive symptoms in wave 3. The number of books at home also significantly predicted higher depressive symptoms in wave 9. Regarding cognition, the number of books at home had a significant direct effect on cognition in wave 3, but an indirect effect on cognition in wave 9 via depression in wave 9. Financial hardship had an indirect effect on cognition in wave 9 via depression in wave 9.

**Conclusion:**

Further analyses are currently being performed, however, preliminary results suggest different associations according to the deprivation variable used and the importance of depressive symptoms as an underlying mechanism. With this study and further analyses, we expect to show the long‐lasting impact of a specific type of adversity (i.e., deprivation) on mental health and cognition while understanding the mechanistic pathway of this impact. Our results aim to inform about the risk factors for later life cognition.

